# From spatial patterning to coordinated execution: a cross-system framework for autism spectrum disorder

**DOI:** 10.3389/fpsyt.2026.1883356

**Published:** 2026-07-06

**Authors:** John Jay Gargus

**Affiliations:** 1 Departments of Pediatrics, Medical Genetics & Genomics, Physiology & Biophysics and Center for Autism Research & Translation, University of California Irvine, Irvine, CA, United States; 2 NeuroQure, Dana Point, CA, United States

**Keywords:** autism spectrum disorder, calcium signaling, cross-system coordination, enteric nervous system, inositol 1,4,5-trisphosphate receptors, neural crest, neural synchronization, planar cell polarity

## Abstract

Autism spectrum disorder (ASD) is characterized by differences in social communication and restricted or repetitive behaviors, yet converging evidence also points to altered neural timing, synchronization, and large-scale coordination despite largely preserved gross neuroanatomy. Parallel observations across non-neural systems, including functional gastrointestinal disturbances, minor anomalies in neural crest–derived tissues, and variation in epithelial patterning outcomes, raise the possibility of a broader vulnerability in coordination-dependent biological processes. Here, we propose a framework in which developmental systems distinguish between spatial patterning mechanisms that establish tissue geometry and a calcium-dependent execution layer that stabilizes coordinated behavior across time. Patterning systems, including planar cell polarity signaling, morphogen gradients, and neural crest guidance cues, specify directional organization and spatial relationships among cells. Their realization, however, requires intracellular processes capable of synchronizing cytoskeletal remodeling, adhesion dynamics, excitability, and metabolic coupling across cells and networks. Within this framework, intracellular Ca²^+^ signaling - and particularly endoplasmic reticulum Ca²^+^ release mediated by inositol 1,4,5-trisphosphate receptors (ITPRs) - represents a candidate mechanism for stabilizing coordinated execution across biological systems. Partial disruption of this coordination layer could degrade synchronization, propagation, and temporal integration without abolishing underlying structural organization. This perspective provides a potential basis for linking altered cortical synchronization with coordination-dependent processes in enteric function, neural crest development, and epithelial patterning outcomes observed in ASD.

This framework is not proposed as a universal explanation for ASD, but as a testable organizing model for coordination-related phenotypes across systems. By distinguishing spatial developmental instruction from the mechanisms that stabilize coordinated execution, this perspective generates experimentally tractable predictions across cellular, organoid, and systems-level models and offers a unifying framework for investigating coordination-dependent biology in ASD.

## Graphical Abstract

**Figure:**
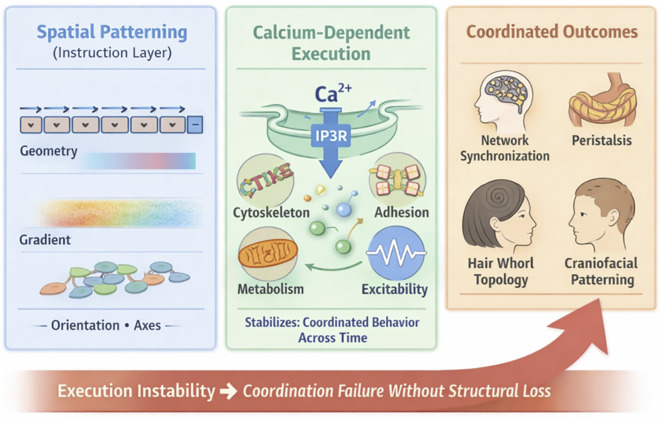
Spatial patterning establishes biological structure; calcium-dependent execution stabilizes coordinated developmental realization across systems. Instability in this execution layer may contribute to coordination-related features in autism spectrum disorder. Developmental patterning systems - including planar cell polarity signaling, morphogen gradients, and neural crest guidance mechanisms - establish tissue geometry and directional organization. The realization of these spatial developmental programs requires a calcium-dependent execution layer that coordinates cytoskeletal remodeling, adhesion dynamics, excitability, and metabolic coupling across cells and tissues. Within the present framework, spatial patterning and coordinated execution represent the mechanistically shared developmental processes that operate across multiple biological systems. The mature functions that emerge from these processes differ among tissues and are therefore not considered part of the proposed unifying mechanism. Perturbation of the execution layer may impair synchronization and temporal stability while preserving underlying structural organization, potentially contributing to coordination-related phenotypes across neural, enteric, neural crest–derived, and epithelial systems in autism spectrum disorder.

## 1 Introduction

Autism spectrum disorder (ASD) is clinically defined by differences in social communication and restricted or repetitive behaviors (1), yet converging evidence also points to alterations in neural timing, synchronization, and large-scale coordination despite largely preserved gross neuroanatomy (2–8). These observations suggest a selective vulnerability in the development of coordinated neural function rather than a primary failure of anatomical patterning alone.

Parallel findings outside the nervous system raise the possibility that this vulnerability extends beyond cortical circuitry. Functional gastrointestinal disturbances, subtle anomalies in neural crest-derived tissues, and variation in epithelial patterning outcomes such as atypical scalp hair whorls and dermatoglyphics have all been reported in subsets of individuals with ASD (3, 9–11). Although heterogeneous and non-diagnostic, these features suggest that coordination-dependent developmental processes across multiple biological systems may be affected.

This apparent dissociation between preserved structure and altered coordination raises a broader biological question: how can complex systems retain spatial organization while exhibiting instability in coordinated function? Here, we propose a framework in which developmental systems comprise two interacting layers: spatial patterning mechanisms that establish tissue geometry and directional organization, and a coordination-dependent execution layer that stabilizes coherent biological behavior across time. Throughout this manuscript, the term “execution” refers to the coordinated realization of previously specified developmental instructions through processes such as cytoskeletal remodeling, adhesion dynamics, migration, excitability, and intercellular synchronization. The mature functions that emerge from these processes differ substantially among tissues and organ systems and are therefore not considered part of the proposed unifying framework. The present model focuses specifically on the shared developmental mechanisms that precede those diverse functional outcomes.

Patterning systems - including planar cell polarity signaling, morphogen gradients, and neural crest guidance cues - define axes, orientation, and positional relationships among cells (12–15). However, realization of these developmental programs additionally requires coordinated cytoskeletal remodeling, adhesion dynamics, and mechanochemical coupling across cells and tissues (16, 17). Intracellular Ca²^+^ signaling is well positioned to support this coordination because it integrates signaling, mechanics, excitability, and metabolism across spatial and temporal scales (18–21).

Within this framework, endoplasmic reticulum Ca²^+^ release mediated by inositol 1,4,5-trisphosphate receptors (ITPRs) represents a key candidate mechanism for stabilizing coordinated execution across biological systems (22, 23). Partial disruption of calcium-dependent coordination could impair synchronization, propagation, and temporal integration without abolishing underlying structural organization. Such a mechanism helps link altered cortical synchronization with coordination-dependent processes in non-neural systems, including enteric function, neural crest development, and epithelial patterning outcomes.

The distinction between spatial instruction and coordinated execution may represent a broader organizational principle across biological development. Systems requiring large-scale synchronization, extended developmental timing, or collective cellular transitions may be particularly sensitive to perturbations affecting execution stability (16–19, 24). Within this perspective, coordination-related features observed in ASD may reflect reduced reliability in the processes that convert spatial developmental programs into coherent biological function rather than a primary disruption of pattern specification itself.

ASD is highly heterogeneous and likely encompasses diverse genetic, epigenetic, metabolic, immunologic, and environmental etiologies. The framework proposed here is therefore not intended as a universal explanation for ASD, nor does it imply that all forms of autism arise through a common biological mechanism. Rather, it identifies a potential coordination-dependent developmental layer through which diverse upstream perturbations may influence developmental outcomes across multiple biological systems.

This framework is intended as an organizational and hypothesis-generating model rather than a claim of a singular causal pathway in ASD. In the sections that follow, we examine the distinction between spatial patterning and coordinated execution as a general developmental principle, explore how this framework may apply across cortical, enteric, neural crest-derived, and epithelial systems, and discuss experimentally tractable predictions arising from this perspective.

## 2 Spatial instruction: patterning systems define biological geometry

Biological systems must establish not only cellular identity but also spatial organization across tissues. During development, this organization is specified through patterning mechanisms that define axes, orientation, and directional relationships among cells (14, 24). Among the best characterized of these systems is planar cell polarity (PCP) signaling, a conserved pathway that coordinates cellular alignment within the plane of an epithelium (12, 25). PCP signaling governs diverse developmental processes including neural tube closure, convergent extension, cochlear hair-cell orientation, and hair follicle alignment (26, 27), underscoring its central role in establishing tissue geometry across vertebrate development.

Related patterning systems, including morphogen gradients and neural crest guidance cues, likewise provide positional information that shapes tissue architecture and migration trajectories (13, 28). Together, these mechanisms establish directional fields that guide how cells orient, move, and interact within developing structures. In this sense, they define the spatial logic of biological systems by specifying where coordinated behavior should emerge and along which axes it should be organized.

Spatial specification alone, however, is insufficient to ensure stable developmental outcomes. A polarity field may be established while the cellular behaviors required to realize that field remain unstable or poorly coordinated (16, 17). Cells must undergo coordinated cytoskeletal remodeling, regulate adhesion dynamics, generate force, and synchronize transitions across neighboring populations to produce stable tissue-level organization (17, 29). These processes unfold across time and depend on reliable coordination across large populations of interacting cells.

Patterning systems therefore define biological geometry but do not by themselves ensure its stable execution. Development additionally depends on mechanisms capable of coordinating cellular behavior across time, particularly in systems requiring collective migration, large-scale alignment, or synchronized activity. The central challenge is therefore not only how biological patterns are specified, but how they are translated into stable and coordinated function.

## 3 Calcium-dependent execution: stabilizing coordinated behavior across time

If spatial patterning systems define the geometry of developing tissues, additional mechanisms are required to translate these instructions into stable biological function across time. This requirement is particularly pronounced in systems that depend on coordinated activity across large populations of cells, where successful development requires not only correct orientation but also reliable execution of collective cellular behaviors. Once established, polarity fields must be realized through coordinated cytoskeletal remodeling, adhesion turnover, force generation, and metabolic support across neighboring cells and tissues (16, 17).

Intracellular Ca²^+^ signaling is well positioned to contribute to this role. Calcium ions function as versatile second messengers that regulate cytoskeletal dynamics, cell adhesion, excitability, secretion, and mitochondrial metabolism (18, 19, 21). Importantly, Ca²^+^ signaling is organized across multiple spatial and temporal scales, ranging from localized microdomains to oscillations and intercellular waves (17, 18, 30). These properties enable coordination within individual cells while supporting synchronized activity across neighboring cells and tissue domains (30, 31).

Within this framework, intracellular Ca²^+^ signaling may function as an execution layer that stabilizes the realization of spatially specified developmental programs. Patterning systems define axes of organization, whereas calcium-dependent dynamics influence whether these programs are executed in a stable and coordinated manner.

This role is particularly evident in systems requiring propagation, synchronization, or collective cellular transitions. Epithelial morphogenesis depends on coordinated cytoskeletal remodeling across contiguous cell sheets (16, 17). Neural systems rely on synchronized activity across distributed networks (32, 33), while contractile tissues require spatiotemporally coordinated activation to generate effective force (34). In each case, disruption of calcium dynamics can degrade coordination without necessarily abolishing underlying structural organization (17, 29).

Taken together, intracellular Ca²^+^ signaling provides a conserved mechanism for stabilizing coordinated behavior across diverse biological systems. Perturbation at this level offers a plausible route through which synchronization, propagation, and temporal integration may become unstable while core spatial organization remains largely intact. Such a distinction is consistent with observations in ASD of altered functional coordination in the context of broadly preserved neuroanatomy.

Calcium signaling is emphasized here not because it is the only mechanism capable of coordinating developmental processes, but because it directly regulates cytoskeletal remodeling, adhesion dynamics, migration, excitability, transcriptional responses, and metabolic coupling. Few signaling systems influence such a broad range of coordination-dependent cellular behaviors across multiple biological scales, making intracellular calcium dynamics a biologically plausible candidate for stabilizing developmental execution.

## 4 ITPR signaling as a molecular fulcrum of the execution layer

If intracellular Ca²^+^ signaling contributes to the stabilization of coordinated biological behavior, an important question is how this regulation is organized to support reliable execution across systems. Among the principal mediators of intracellular calcium dynamics are inositol 1,4,5-trisphosphate receptors (ITPRs), which control Ca²^+^ release from the endoplasmic reticulum and shape intracellular calcium oscillations, waves, and microdomains (22, 23, 35).

ITPR-mediated Ca²^+^ release occupies a strategic position within the cell by integrating membrane-derived signals with intracellular calcium stores to generate temporally structured signaling dynamics (19, 36). These dynamics regulate processes central to coordinated cellular function, including cytoskeletal remodeling, adhesion turnover, excitability, and metabolic coupling through interactions with mitochondria (17, 19, 37). Because these functions operate at the interface of signaling, mechanics, and energy supply, ITPRs are well positioned to influence the temporal stability of cellular behavior across tissues and networks.

Within this framework, ITPR signaling contributes primarily to execution stability rather than spatial pattern specification itself. Systems requiring synchronized transitions across large populations of cells may therefore be particularly sensitive to perturbations in ITPR-mediated Ca²^+^ dynamics, where even modest alterations could destabilize coordination without disrupting underlying structural organization.

An illustrative example is provided by the BTBR *T^+^Itpr3^tf^*/J mouse strain, in which a mutation affecting Itpr3 alters intracellular Ca²^+^ signaling without directly disrupting canonical planar polarity pathways (38–40). This strain exhibits atypical epithelial patterning, gastrointestinal dysfunction, immune dysregulation, and behavioral phenotypes relevant to ASD. In addition, a gain-of-function mutation within the regulatory domain of Itpr3 alters calcium signaling and taste receptor transduction, producing altered taste preference as a functional readout of its causal role (41). Although no single model establishes a general developmental principle, this convergence supports the possibility that altered calcium-dependent signaling may contribute to coordination-related phenotypes across multiple systems in the context of largely preserved structure.

Taken together, these observations position ITPR-mediated Ca²^+^ signaling as a potential point of convergence for coordination-dependent developmental processes. Vulnerability at this level could propagate across systems that rely on synchronized cellular behavior, linking diverse phenotypes without requiring a shared defect in spatial patterning. Calcium signaling is emphasized here not as a uniquely causal mechanism, but as a biologically well-characterized system capable of integrating cytoskeletal, metabolic, and excitatory processes across multiple organizational scales.

## 5 Hair whorls as developmental readouts of pattern execution

Epithelial patterning provides an accessible example of how spatial developmental programs are translated into stable biological outcomes. In the scalp, hair follicle orientation is established during a restricted window of fetal development through planar patterning mechanisms that align epithelial cells across a large tissue field (25, 42). Similar principles extend beyond scalp patterning alone. Dermatoglyphic ridge patterns are likewise established during early fetal development through coordinated epithelial growth dynamics. Once established, these patterns are maintained throughout life, making scalp hair and dermatoglyphic topology durable records of early morphogenetic processes.

Within this framework, epithelial patterning outcomes may reflect not only spatial developmental instruction but also the stability with which these programs are executed during development. Planar cell polarity and related signaling systems define the directional organization of follicle growth (25, 26, 43), yet realization of this organization requires coordinated cellular behavior across the epithelium, including cytoskeletal remodeling, adhesion dynamics, and distributed mechanical interactions (16, 17). The resulting pattern therefore reflects both underlying positional information and the reliability of coordinated cellular execution.

If calcium-dependent signaling contributes to this execution layer, partial disruption of coordinated cellular behavior could alter epithelial patterning outcomes without disrupting overall tissue structure. Such perturbations may manifest as variation in pattern topology, including differences in follicle orientation or spatial arrangement, reflecting variability in how polarity fields are resolved rather than failure of pattern specification itself.

This perspective may be relevant to ASD, where differences in scalp hair whorl and dermatoglyphic topology have been reported at the group level relative to controls (11, 44–48). These findings are non-diagnostic, heterogeneous, and incompletely characterized, but may be consistent with altered coordination-dependent developmental processes. Epithelial patterning therefore provides a potential example of how early variation in the execution of spatial developmental programs may persist as stable anatomical “fossil” outcomes. Importantly, these observations remain preliminary and require systematic validation across larger and more diverse populations.

More broadly, epithelial systems illustrate a general developmental principle: spatial organization may be established while the stability of coordinated execution varies. Patterning outcomes may therefore preserve signatures of how reliably coordinated cellular behavior emerged during development, providing a potential bridge between cellular patterning mechanisms and system-level coordination.

## 6 Cross-system parallels: coordination across brain, gut, and neural crest

The distinction between spatial instruction and coordinated execution extends across multiple biological systems that must translate spatial organization into temporally stable function (16, 17). Although these systems differ in structure and physiological role, they share a common requirement: reliable coordination of activity across cells and networks distributed in space and time. In the cerebral cortex, large-scale association networks must synchronize activity across distributed regions to support integrative cognition (32, 49). Anatomical architecture, organization and connectivity provides the structural framework for these interactions (50), but functional integration depends on precise temporal coordination across neuronal populations (33).

In ASD, alterations in network synchronization and temporal integration have been reported despite broadly preserved cortical structure (2, 7, 8), consistent with reduced stability in coordinated neural execution.

A related principle is evident in the gastrointestinal system, where directional organization must be translated into effective propagation of activity along the gut. Peristalsis depends on coordinated interactions among smooth muscle, enteric neurons, and pacemaker cells, requiring temporally structured activation to generate effective motility (9, 34, 51). Functional gastrointestinal disturbances frequently reported in ASD, often in the absence of consistent structural abnormalities, are similarly compatible with disrupted coordination rather than failed tissue specification (52).

Neural crest development provides an example at an earlier developmental stage. Neural crest cells migrate collectively through processes requiring coordinated polarity, cytoskeletal remodeling, adhesion dynamics, and temporally regulated signaling (13, 53). Their derivatives include craniofacial structures, melanocytes, peripheral nerves, and components of the enteric nervous system. Subtle anomalies observed in ASD, including minor craniofacial differences, may therefore reflect variation in developmental execution rather than major defects in tissue specification (11, 44, 53).

Across these systems, phenotypes are characterized less by loss of structure than by altered synchronization, propagation, or topological organization within otherwise intact architectures. This convergence suggests that coordination-dependent biological processes may share sensitivity to perturbations affecting mechanisms that stabilize execution across time.

Within this framework, cortical synchronization, enteric motility, neural crest migration, and epithelial patterning can be understood as distinct manifestations of a broader developmental constraint. Systems requiring large-scale integration, extended temporal regulation, or collective cellular behavior may be particularly vulnerable to instability in mechanisms supporting coordinated execution. Importantly, this perspective does not require identical molecular mechanisms across systems, but rather a shared sensitivity to perturbations affecting the stability of biological coordination.

## 7 ASD as a disorder of pattern stabilization under constraint

Within this framework, ASD can be understood as reflecting vulnerability in mechanisms that stabilize coordinated biological behavior across time. In this context, biological constraints refer to factors that limit or channel developmental outcomes, including signaling dynamics, energetic requirements, developmental timing, and cellular coordination demands. The central issue is not necessarily disruption of spatial developmental patterning itself, but reduced reliability in the execution of those patterns in systems that depend on precise temporal coordination across cells and networks. This perspective is consistent with observations that gross anatomical organization is largely preserved in ASD, while functional measures reveal alterations in timing, synchronization, and integration (2, 6, 7).

This distinction helps reconcile findings across multiple biological systems. In the brain, alterations in large-scale synchronization and temporal integration occur in the context of intact cortical organization (2, 8). In the gastrointestinal system, functional disturbances are common despite the absence of a defining structural lesion (52). Minor physical anomalies, including neural crest-derived features and epithelial patterning variation, are likewise more consistent with altered developmental execution than with major defects in tissue specification (11, 45). Across these domains, phenotypes are characterized less by loss of structure than by variability in the stability and coordination of biological activity.

Recent iPSC-derived neuronal and cerebral organoid models of ASD and related neurodevelopmental conditions have demonstrated altered neuronal maturation, impaired synchronization, hyperexcitability, and asynchronous developmental trajectories despite broadly preserved tissue organization (54–58). These findings are consistent with the possibility that coordination-dependent developmental processes may become unstable without requiring major disruption of underlying anatomical patterning. Such systems provide emerging experimental platforms for investigating how intracellular signaling dynamics influence execution stability across developing neural networks.

Within this perspective, ASD may be viewed as a condition in which coordination-dependent systems operate under reduced developmental stability or increased biological constraint. Systems requiring extended developmental timing, high metabolic demand, or large-scale synchronization may be particularly sensitive to perturbations affecting mechanisms that support coordinated execution, including calcium-dependent signaling (17, 29). Modest alterations in intracellular ITPR signaling dynamics, as reported in ASD patient-derived cells (59–61), could bias development toward variability in synchronization, propagation, or topological resolution without disrupting the underlying spatial framework.

Importantly, this framework does not imply a single causal mechanism for ASD, nor that calcium signaling alone accounts for phenotypic diversity (5, 10). Rather, it identifies a potential shared point of vulnerability: the stabilization of coordinated biological behavior required to reliably realize spatial developmental programs. Diverse genetic, epigenetic, metabolic, and environmental factors may converge on this execution layer, producing variability in coordination rather than uniform structural disruption.

Direct mechanistic links between intracellular calcium dynamics and large-scale circuit dysfunction in ASD remain incompletely defined and represent an important area for future investigation (41, 54–56, 58, 62).

## 8 Empirical ground, limitations, and testable predictions

Several components of the framework proposed here are grounded in established biological principles. Spatial patterning systems - including planar cell polarity signaling, morphogen gradients, and neural crest guidance cues - define tissue geometry and directional organization across developmental contexts. Intracellular Ca²^+^ signaling regulates cytoskeletal dynamics, adhesion, excitability, and metabolic coupling, and contributes to the coordination of cellular behavior across tissues and networks. In parallel, ASD has been associated with alterations in neural timing and synchronization, functional gastrointestinal disturbances, and minor physical anomalies. Together, these observations support the plausibility of a framework linking coordination-dependent biological processes across systems.

At the same time, the central integrative claims remain hypothetical, and direct cross-scale causal relationships between intracellular Ca²^+^ dynamics and system-level coordination phenotypes have not yet been established. In particular, it remains unclear whether variation in epithelial patterning outcomes directly reflects altered calcium-dependent developmental execution, or whether intracellular Ca²^+^ dynamics provide a common mechanistic substrate linking neural and non-neural systems in ASD (58, 59).

Model systems such as the BTBR *T^+^Itpr3^tf^*/J mouse provide evidence linking altered ITPR signaling to intracellular Ca²^+^ dysregulation and coordination-related phenotypes across multiple systems. However, it remains uncertain whether epithelial patterning reliably indexes coordination dynamics in cortical or enteric systems, or whether ITPR-mediated signaling abnormalities are sufficient to account for broader ASD-related phenotypes. Alternative or additional mechanisms - including synaptic, metabolic, electrophysiological, or network-level processes - may also contribute to the stabilization of coordinated biological behavior.

The proposed framework therefore should be understood as a testable organizing model rather than a definitive explanation for ASD. Its value lies in generating experimentally tractable questions regarding the relationship between developmental patterning, intracellular signaling dynamics, and system-level coordination.

Several predictions follow from this perspective. First, perturbations in intracellular Ca²^+^ signaling should preferentially disrupt coordination-dependent processes while preserving core spatial patterning architecture. Second, systems requiring large-scale synchronization or collective cellular behavior should exhibit parallel signatures of reduced temporal stability across neural and non-neural domains. Third, coordination-related functional differences may emerge before overt structural abnormalities become apparent during development. Finally, if epithelial patterning outcomes reflect variation in developmental execution, these features may correlate with independent measures of neural or physiological coordination.

**Figure 1:**
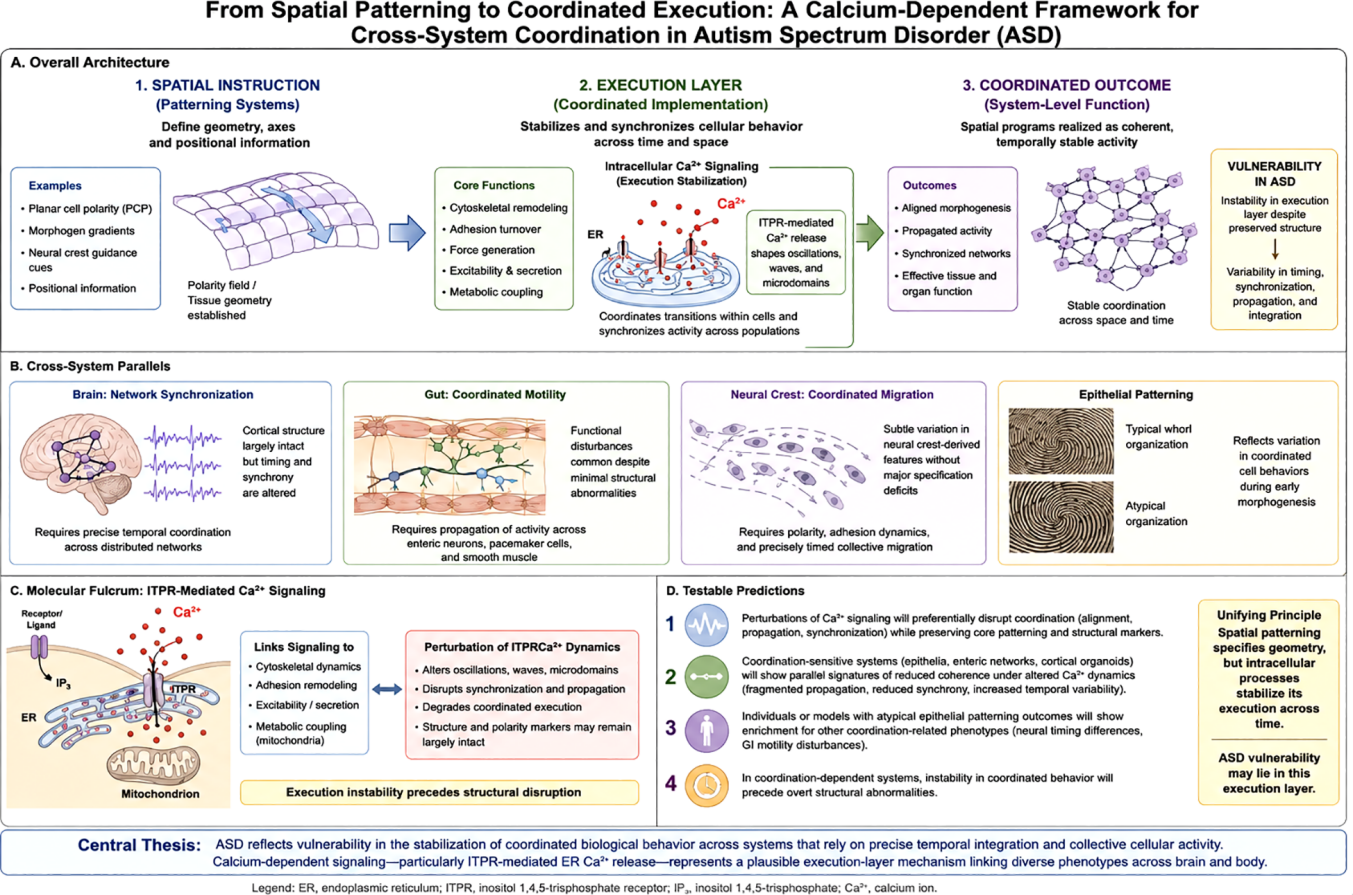
Spatial patterning and calcium-dependent execution across biological systems. **(A)** Developmental patterning systems, including planar cell polarity (PCP) signaling, morphogen gradients, and neural crest guidance cues, establish tissue geometry and directional organization. **(B)** Intracellular Ca²^+^ signaling, particularly endoplasmic reticulum Ca²^+^ release mediated by ITPRs, contributes to the stabilization of cellular behavior through effects on cytoskeletal dynamics, adhesion, excitability, and metabolic coupling. **(C)** Interaction between spatial patterning and calcium-dependent execution supports stable biological outcomes across tissues, including epithelial alignment, synchronized neural activity, coordinated contractile propagation, and collective cell migration. **(D)** Coordination-dependent processes across brain, gut, neural crest-derived tissues, and epithelia share sensitivity to perturbations affecting temporal stability and synchronization. **(E)** Partial disruption of calcium-dependent execution may impair synchronization, propagation, or integration without abolishing underlying structural organization, producing variability across coordination-dependent systems in ASD. **(F)** The framework predicts that perturbations affecting intracellular Ca²^+^ dynamics will preferentially disrupt coordination-dependent processes while preserving core spatial patterning architecture.

The framework (**Figure 1**) would be challenged by evidence demonstrating intact coordination despite substantial disruption of intracellular calcium dynamics, or by coordination-related phenotypes arising independently of mechanisms affecting cellular synchronization and temporal stability. Clarifying how intracellular signaling dynamics influence coordination across biological scales therefore remains an important direction for future investigation (61, 62).

## Discussion

Biological systems appear to follow a recurring organizational principle in which spatial patterning mechanisms establish structure, while intracellular processes stabilize the coordinated execution of that structure across time. This distinction provides a framework for understanding how tissues and networks can retain anatomical organization while exhibiting variability in synchronization, propagation, and coordinated function (**Box 1**).

Box 1 **Spatial Patterning and Coordinated Execution as Distinct Developmental Layers**

Biological systems must establish both spatial organization and stable function across time. Developmental patterning mechanisms - including planar cell polarity signaling, morphogen gradients, and neural crest guidance cues - define tissue geometry, orientation, and positional relationships among cells. However, realization of these spatial programs additionally requires mechanisms that stabilize coordinated cellular behavior across tissues and networks.

Processes such as cytoskeletal remodeling, adhesion dynamics, excitability, and metabolic coupling must be integrated across populations of cells to generate stable biological function. Intracellular signaling systems, including calcium-dependent pathways, are well positioned to support this coordination by linking signaling dynamics with mechanical, metabolic, and electrophysiological processes.

Within this framework, disruption of coordinated execution may impair synchronization, propagation, or temporal stability without abolishing underlying structural organization. This distinction provides a potential basis for understanding how coordination-related phenotypes can emerge across systems that retain broadly intact anatomy.

More broadly, the transition from spatial organization to coordinated function may represent a recurring organizational principle across biological development and physiology.

Within this perspective, ASD may reflect vulnerability in mechanisms that stabilize coordinated biological behavior rather than disruption of developmental patterning itself. Systems that depend on precise temporal synchronization, large-scale integration, or collective cellular dynamics may be particularly sensitive to perturbations affecting coordinated execution. The resulting phenotype is therefore characterized less by loss of structure than by variability in timing, integration, and functional coherence across otherwise intact systems.

This model integrates observations across multiple levels of biology, including cortical network synchronization, enteric function, neural crest-derived features, and epithelial patterning outcomes. Intracellular Ca²^+^ signaling - particularly endoplasmic reticulum Ca²^+^ release mediated by ITPR - represents a plausible component of this execution layer, linking cytoskeletal dynamics, metabolic coupling, excitability, and intercellular coordination across tissues (22, 35, 37).

Importantly, this perspective is intended as a testable organizing model rather than a universal explanation for ASD. Its value lies not in proposing a single causal mechanism, but in identifying a potential shared point of vulnerability across systems that depend on reliable coordination over time. By distinguishing between the specification of spatial pattern and the stabilization of its execution, this framework provides a basis for integrating diverse observations while generating experimentally tractable hypotheses across biological scales.

Although ASD is diagnostically defined by behavioral criteria, accumulating evidence suggests that coordination-related features may extend beyond the nervous system to include gastrointestinal, developmental, and other somatic manifestations. Within the present framework, these features are viewed not simply as independent comorbidities, but as potentially reflecting shared vulnerabilities in coordination-dependent biological processes across systems.

More broadly, the distinction between spatial organization and coordinated execution highlights a general principle of biological systems: the transformation of structure into stable function across time. ASD may therefore be understood, in part, as reflecting altered stability in this transformation across coordination-dependent systems.
